# Human growth factor-mediated signalling through lipid rafts regulates stem cell proliferation, development and survival of *Schistosoma mansoni*

**DOI:** 10.1098/rsob.230262

**Published:** 2024-01-10

**Authors:** Shradha Maharjan, Ruth S. Kirk, Scott P. Lawton, Anthony J. Walker

**Affiliations:** ^1^ Molecular Parasitology Laboratory, School of Life Sciences, Pharmacy and Chemistry, Kingston University, Penrhyn Road, Kingston upon Thames KT1 2EE, UK; ^2^ Centre for Epidemiology and Planetary Health, SRUC School of Veterinary Medicine, Scotland's Rural College, West Mains Road, Edinburgh EH9 3JG, UK

**Keywords:** schistosome, lipid rafts, schistosomiasis, protein kinase, epidermal growth factor receptor, insulin receptor

## Abstract

Although the mechanisms by which schistosomes grow and develop in humans are poorly defined, their unique outer tegument layer, which interfaces with host blood, is considered vital to homeostasis of the parasite. Here, we investigated the importance of tegument lipid rafts to the biology of *Schistosoma mansoni* in the context of host–parasite interactions. We demonstrate the temporal clustering of lipid rafts in response to human epidermal growth factor (EGF) during early somule development, concomitant with the localization of anteriorly orientated EGF receptors (EGFRs) and insulin receptors, mapped using fluorescent EGF/insulin ligand. Methyl-*β*-cyclodextrin (M*β*CD)-mediated depletion of cholesterol from lipid rafts abrogated the EGFR/IR binding at the parasite surface and led to modulation of protein kinase C, extracellular signal-regulated kinase, p38 mitogen-activated protein kinase and Akt signalling pathways within the parasite. Furthermore, M*β*CD-mediated lipid raft disruption, and blockade of EGFRs using canertinib, profoundly reduced somule motility and survival, and attenuated stem cell proliferation and somule growth and development particularly to the fast-growing liver stage. These findings provide a novel paradigm for schistosome development and vitality in the host, driven through host–parasite interactions at the tegument, that might be exploitable for developing innovative therapeutic approaches to combat human schistosomiasis.

## Graphical abstract

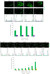

## 1. Introduction

*Schistosoma* blood flukes cause human schistosomiasis, a major neglected tropical disease (NTD) of global significance with approximately 240 million people infected mostly across low- to middle-income countries [1,2]. When schistosomes penetrate the skin, they transform rapidly from a free-living cercaria to a parasitic schistosomulum (also known as a somule) [3]. The somules then embark on an extraordinary journey, first accessing the bloodstream by penetrating dermal blood vessels, and then migrating to the branches of the hepatic portal vein after passing through the heart and lungs [4]. Upon arrival at the liver, the somules ingest blood cells and grow exponentially, doubling in weight approximately every 2.3 days [5]. Next, the adult male and female worms couple, mature and journey to their preferred mating sites, the mesenteric veins (*Schistosoma mansoni* and *S. japonicum*), or the veins surrounding the bladder (*S. haematobium*), where they lay hundreds to thousands of eggs daily for disease transmission [6]. Schistosome eggs that fail extravasation and host exit get trapped in tissues such as the gut, liver, and spleen and release immunomodulatory molecules that drive the generation of granulomas and fibrosis, pathologies associated with the disease [7,8].

During cercaria to somule transformation, the schistosome undergoes significant physiological and biochemical change and develops a tegument that matures as the parasite develops and which remains into adulthood [3,9–11]. In direct contact with host blood, this unique, specialized organ, which comprises a syncytial cytoplasm overlaid with double-bilayer membrane, supports vital parasite processes such as osmoregulation, blood glucose uptake and host immune evasion [12–15]. Many potential anti-schistosome vaccine targets, such as tetraspanins and Smp80/calpain also reside in the outer membrane layer [16,17], signifying its importance to parasite survival.

Lipid rafts are small microdomains present in cell surface membranes that are rich in cholesterol and sphingolipids, cholesterol being the main component [18]. These microdomains are dynamic in nature, exist in discrete liquid ordered form and are more tightly packed than the surrounding non-raft membrane regions [18,19]. Upon cell stimulation, the lipid rafts cluster and serve as platforms for various cellular functions including protein sorting, membrane trafficking and intracellular signalling [19–22]. While certain proteins such as flotillins are permanently associated with raft microdomains [23], some are transiently associated; for example, certain growth factor receptors, other tyrosine kinase receptors, mitogen-activated protein kinases (MAPKs) and protein kinase C (PKC) are predominantly found in or are associated with lipid rafts [21]. Lipid rafts have been studied in several unicellular parasites including *Plasmodium* spp. [24–26], *Leishmania* spp. [27] and *Trypanosoma* spp. [28]; however, these microdomains have been seldom studied in relation to communication from the host to the parasite, with an even greater lack of knowledge evident for multicellular parasites including schistosomes. The lack of detailed understanding of these microdomains in schistosomes remains a significant impediment to understanding the parasite's biology given the potential for the rafts to be involved in host growth-factor to parasite communication and function-dependent downstream signalling within the schistosome.

Here, employing *S. mansoni* somules, we aimed to characterize in detail the importance of tegument lipid rafts to schistosome biology in the context of host–parasite interactions. We demonstrate human epidermal growth factor (EGF)-mediated clustering of lipid rafts, map EGF and insulin binding at parasite surface, and define the importance of tegument cholesterol to cellular signal transduction events in the parasite. Moreover, we demonstrate that lipid raft disruption, and inhibition of EGF receptor (EGFR) using canertinib, profoundly attenuate somule stem cell proliferation and somule growth and development. Collectively, the results of this study provide novel insights into human–schistosome host–parasite interactions, that have relevance to other host–parasite systems, and that could underpin efforts to develop new therapeutic approaches to combat this devastating NTD.

## 2. Results

### 2.1. Lipid raft formation is anteriorly orientated in early developing *S. mansoni* somules

**Figure 1.**
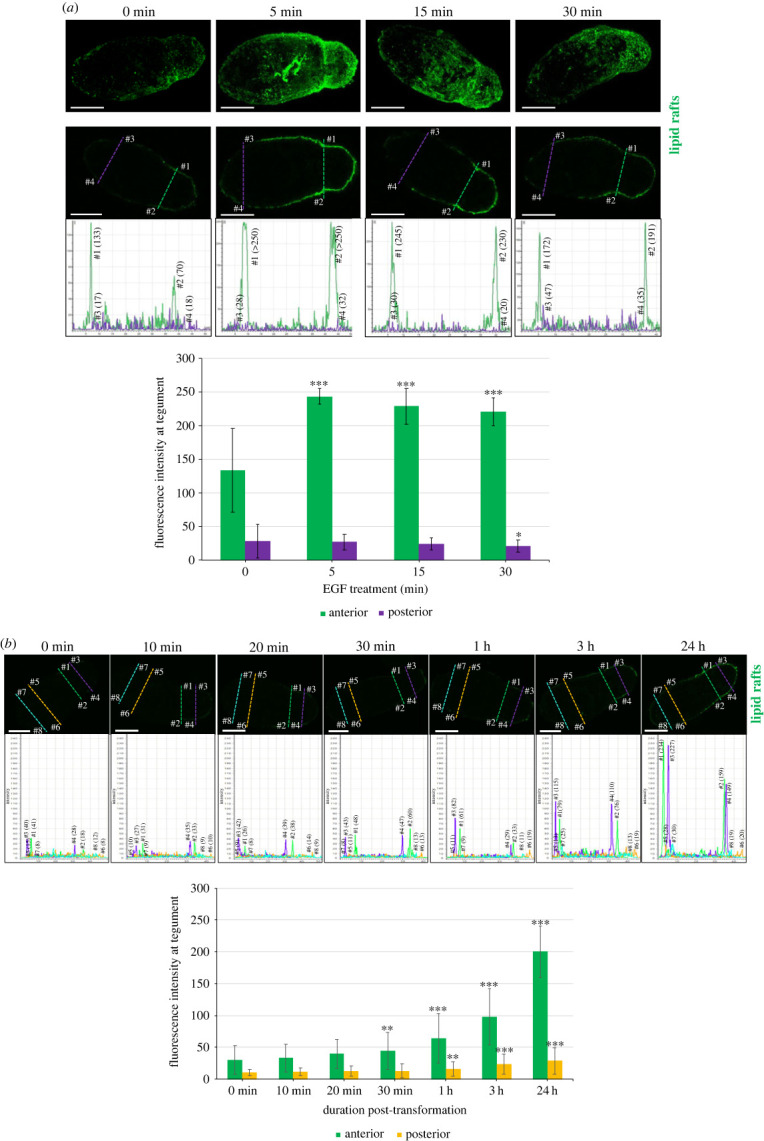
EGF-mediated lipid raft formation is anteriorly oriented in developing *S. mansoni* somules. (*a*) *In vitro* transformed 1-day-old somules were either left untreated (0 min) or were treated with human EGF (15 ng ml^−1^) for increasing durations prior to staining with the Vybrant 488 (green) lipid raft labelling kit and imaging by CLSM. Representative (upper) images are *z*-axes maximum projections of the somules. Fluorescence intensity at the somule tegument (locations: anterior #1, #2; posterior #3, #4) of single *z*-sections from the centre of the parasite (lower images) was determined and the mean ± s.d. fluorescence of the stained lipid rafts calculated (graph). (*b*) Transforming somules (0 min to 24 h) were treated with 15 ng ml^−1^ EGF for 5 min prior to lipid raft labelling (green). Fluorescence intensities at the tegument (locations: anterior #1–#4; posterior #5–#8) from single z-sections from the centre of the parasites were determined and the mean ± s.d. fluorescence of the stained lipid rafts calculated (graph). *p ≤ 0.05, **p ≤ 0.01 and ***p ≤ 0.001, when compared with 0 min; *n* = 20 parasites per treatment. Bars = 25 µm.

Lipid raft microdomains are characterized by their insolubility in non-ionic detergents and presence of the pentasaccharide ganglioside_GM1_. The presence of specialized raft microdomains at the schistosome surface was originally postulated based on lipid content and resistance to detergents [29,30], and more recently confirmed [31], with our previous work indicating that lipid rafts might represent a platform for schistosome host–parasite communication [31]. To characterize in detail the formation of these microdomains in developing *S. mansoni*, *in vitro* cultured 1-day-old somules were treated with human EGF (15 ng ml^−1^), lipid rafts stained with the Vybrant lipid raft staining kit that incorporates fluorescent cholera toxin subunit B (CT-B) that binds ganglioside_GM1_, and parasites visualized by confocal laser scanning microscopy (CLSM). Although GM1 clusters, characteristic of lipid rafts, were apparent under basal (unexposed) conditions, 5 min EGF treatment resulted in enhanced raft clustering (*p* ≤ 0.001) at the anterior of the parasite, with clustering still evident at 30 min (figure 1*a*). Somules not incubated in CT-B (negative controls) showed no fluorescence.

Because cercariae transform rapidly into somules and remodel their surface after penetrating host skin, forming a new multilaminate tegument [9,10], we hypothesized that the tegument might become receptive to host factors during transformation and thus investigated newly transforming somules. Rafts did not appear to form in response to EGF at 10 min or 20 min post-transformation; however, at 30 min, significantly enhanced anteriorly orientated tegument fluorescence was observed (*p* ≤ 0.01; figure 1*b*), supporting the early presence of EGFRs and rafts at the surface of the transforming parasite. The concentration of these rafts increased significantly with development, with a 6.7-fold increase in fluorescence seen at 24 h (*p* ≤ 0.001). The ability of rafts to form in response to EGF seems to develop in an anterior-to-posterior fashion, although posterior raft formation was always substantially lower than that at anterior over 24 h (figure 1).

### 2.2. Human EGF and insulin preferentially bind receptors located at the anterior of developing *S. mansoni* somules

**Figure 2.**
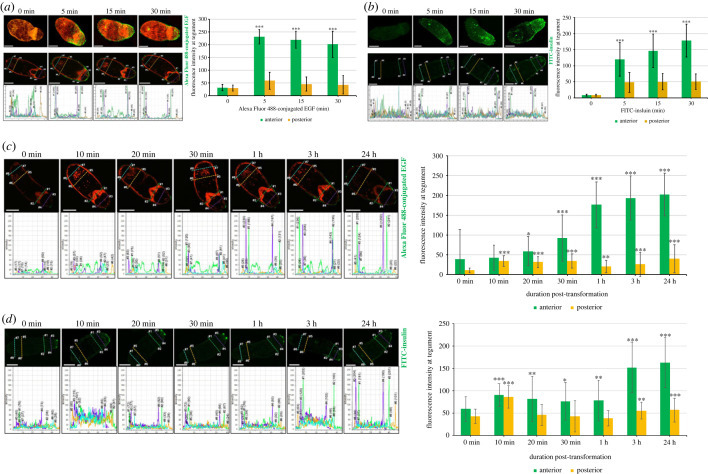
Human EGF and insulin preferentially bind receptors located at the anterior of developing *S. mansoni* somules. (*a,b*) *In vitro* transformed 1-day-old somules were either left untreated (0 min) or were exposed to AlexaFluor 488-conjugated EGF (2 µg ml^−1^; green) or FITC-conjugated insulin (1 µM; green) for increasing durations. Representative (upper) images are *z*-axes maximum projections of the somules. Fluorescence intensity at the somule tegument (locations: anterior #1–#4; posterior #5–#8) of single *z*-sections from the centre of the parasite (lower images) was determined and the mean ± s.d. fluorescence of the ligand calculated (graph). (*c,d*) Transforming somules (0 min to 24 h) were exposed to AlexaFluor 488-conjugated EGF (2 µg ml^−1^; green) or FITC-conjugated insulin (1 µM; green) for 5 min. Fluorescence intensities at the tegument (locations: anterior #1–#4; posterior #5–#8) from single *z*-sections from the centre of the parasite were determined and the mean ± s.d. fluorescence of the bound ligand calculated (graph). (*a,b*) ***p ≤ 0.001, when compared with posterior signal; (*c,d*) *p ≤ 0.05, **p ≤ 0.01 and ***p ≤ 0.001, when compared with 0 min; *n* = 20 parasites per treatment. Parasites stained with AlexaFluor 488-conjugated EGF were also stained with rhodamine phalloidin (red) to visualize actin. Bars = 25 µm.

Because schistosomes possess three EGFRs [32,33], we next mapped, indirectly, surface receptors of human EGF in somules using fluorescently conjugated human EGF as a marker. To determine kinetics of binding, somules were incubated with fluorescent EGF for increasing durations; EGFR binding was seen at the surface and acetabulum of the parasite, with binding preferentially occurring anteriorly as early as 5 min and sustained over 30 min (*p* ≤ 0.001; figure 2*a*), with some posterior binding also evident when compared with controls (*p* ≤ 0.01). The punctate staining (figure 2*a*) indicates that the receptors might localize to lipid rafts. In newly transforming somules, enhanced binding was visible soon after parasite transformation, particularly at the tip of the cone up to 1 h post-transformation (figure 2*c*); thereafter, somules displayed enhanced staining, particularly anteriorly. Analysis of fluorescence intensity revealed significantly increased binding at 20 min and thereafter, with an approximately 5.2-fold increase over control (0 min) at 24 h. Thus proteins, presumably *S. mansoni* EGFRs capable of binding human EGF, are preferentially expressed at the surface of the parasite anteriorly, concomitant with the lipid rafts, which could result from tegument remodelling during the transition from a cercaria to somule.

As insulin receptors (IRs) can localize to lipid rafts in mammalian cells [34] and because *S. mansoni* possesses two IRs [32,33], FITC-conjugated insulin was employed to indirectly localize IRs in somules. IR binding was evident at the surface of 1-day-old somules with negligible fluorescence apparent on somules not exposed to FITC-insulin (figure 2*b*). As with EGF, FITC-insulin primarily bound anterior regions (*p* ≤ 0.001, compared to posterior), including the anterior cone, but also bound to the rear of the parasite. During early transformation, significant binding of FITC-insulin to the parasite surface was evident after 10 min, with anterior staining increasing over time and a 2.6-fold increase seen at 3 h (*p* ≤ 0.001), supporting the increased presence of IRs during transformation (figure 2*d*). Competition assays with saturating concentrations of unconjugated EGF or insulin demonstrate the specificity of the staining (electronic supplementary material, figure S1).

Interrogation of publicly available gene expression data for *S. mansoni* revealed that two of the three EGFRs (Smp_165470, Smp_093930) are highly expressed in the 3 h somule relative to other life stages, whereas the remaining EGFR (Smp_152680 (Smp_344500)) displayed lower relative expression (electronic supplementary material, figure S2*a*). Both IRs are also expressed in somule stage. Moreover, screening of *S. mansoni* single-cell RNA-seq adult worm data available at SchistoCyte Atlas revealed that all five receptors are expressed in the neoblast stem cell populations and the progenitors, including those of the tegument (electronic supplementary material, figure S2*b*); although single-cell RNA-seq data are not available for the other parasite life stages including somules, these data highlight the importance of the EGFRs and IRs in the tegument layer.

### 2.3. Further characterization of lipid rafts in *S. mansoni* somules

**Figure 3.**
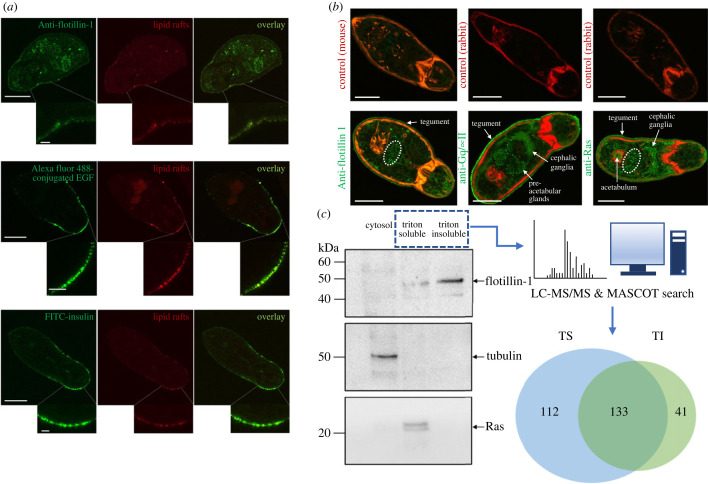
Further characterization of lipid rafts in *S. mansoni* somules. (*a*) EGFRs and IRs colocalize with lipid rafts. One-day-old somules were treated with 15 ng ml^−1^ EGF, stained with the Vybrant 594 (red) lipid raft labelling kit, then stained with AlexaFluor 488-conjugated EGF (2 µg ml^−1^) or FITC-conjugated insulin (1 µM) and imaged by CLSM; the inset panels display higher magnifications of the tegument in the anterior cone region. Representative images are single *z*-sections through the centre of the parasite. Bars = 25 µm. (*b*) *In situ* localization of lipid raft-associated proteins in somules. One-day old somules were fixed and processed for immunofluorescence using anti-flotillin-1, -Gq, or -Ras primary and AlexaFluor 488 (green) secondary antibodies. Rhodamine phalloidin was used to stain F-actin and images were captured by CLSM. Negative control somules (upper panels) displayed little to no background fluorescence. Flotillin-1 localized to the tegument (and with tegument raft clusters, ‘a’) and a group of cells (encircled), whereas Gq and Ras localized to the tegument but also to additional internal structures and cephalic ganglia; Gq was also present in the pre-acetabular glands. Representative micrographs of somules are shown as single *z* sections through the parasite. Bars = 25 µm. (*c*) Triton-insoluble (detergent-resistant) membranes (DRMs) were isolated from 1-day-old somules (approx. 150 000) and the resultant cytosolic, triton-soluble (TS) and triton-insoluble (TI) membrane fractions processed for western blotting with anti-flotillin-1, -Ras and -β-tubulin antibodies. The TS and TI fractions were processed for LC-MS/MS to identify proteins in the individual fractions (pie chart).

Having demonstrated that human EGF and insulin bind the somule surface, their possible localization to lipid rafts was explored. CLSM revealed that the surface distribution of EGFRs and IRs at the anterior cone mirrored that of the lipid rafts, with overlays revealing striking signal co-localization, and high magnification imaging revealing their punctate staining (figure 3*a*). These data support that EGFRs and IRs localize to the GM1 clustered lipid raft microdomains in *S. mansoni* somules. Antibodies against flotillin-1, Gq and Ras, previously validated by us for use in *S. mansoni* [31] and that can be used as lipid raft markers, were next employed to localize these proteins in 24 h somules. All three proteins were clearly expressed at the parasite tegument, with flotillin-1 and Ras also evident in cell clusters proximal to the acetabulum, and Gq and Ras localized to internal structures including the cephalic ganglia (figure 3*b*).

The partitioning of a protein in a detergent-resistant membrane (DRM) preparation indicates that it is raftophilic [35]. Thus, DRMs were prepared from the teguments of 24 h somules for analysis. Western blotting revealed that flotillin-1, a common lipid raft marker, was enriched in the triton-insoluble (TI) DRM fraction (figure 3*c*). Although Ras was apparent in the triton-soluble (TS) fraction, this could be because not all Ras isoforms partition into raft domains [36]; β-tubulin, a cytosolic marker, was exclusively found in the cytosol as expected (figure 3*c*). Somule teguments were next prepared by freeze fracture and separated into TI and TS fractions. Liquid-chromatography mass spectrometry/mass spectrometry (LC-MS/MS) identified 286 proteins, 133 of which were in both TS and TI fractions (TSI), 112 exclusively in the TS fraction, and 41 in the TI fraction alone (figure 3*c*; electronic supplementary material, table S1). Among those proteins present exclusively in the ‘raftophilic’ TI fraction, ten remain uncharacterized (electronic supplementary material, table S1). As expected, the raft marker proteins flotillin-1 (Smp_016200) and flotillin-2 (Smp_033970) were both present only in the TI fraction, highlighting the success of the separation. Other proteins present in the TI fraction included paramyosin (Smp_046060), nebulin (Smp_151490), leishmanolysin-like peptidase (Smp_204600, Smp_171320), low-density lipoprotein (LDL) receptor (Smp_020550), annexin (Smp_045500), GTP-binding nuclear protein (Ran) (Smp_079430), Eps-8-related protein (Smp_139520), calmodulin (Smp_032970), Kunitz-type protease inhibitor (Smp_179120), dynein light chain (Smp_019310), intermediate filament proteins (Smp_170930), myosin light chain 1 (Smp_045220), thioredoxin (Smp_008070) and synaptotagmin (Smp_175900). Surprisingly, linker histone H1 (Smp_162370), histone H2B (Smp_108390) and histone H3 (Smp_074610) were also present (electronic supplementary material, table S1).

### 2.4. Disruption of lipid rafts modulates multiple signalling pathways in *S. mansoni* somules

**Figure 4.**
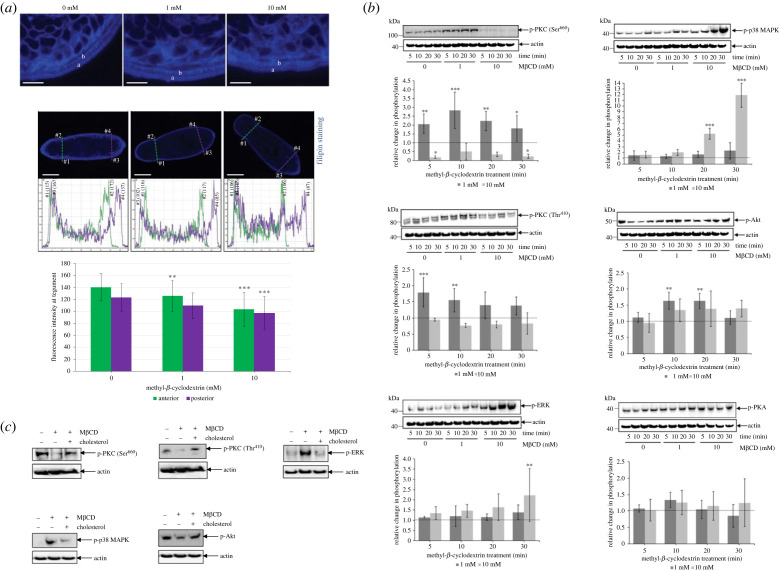
Cholesterol depletion modulates multiple signalling pathways in *S. mansoni* somules. (*a*) One-day-old somules were left untreated (control, 0 mM) or were treated with 1 mM or 10 mM M*β*CD for 30 min (upper panels) or 60 min (lower panels), stained with filipin (blue) and imaged by CLSM; ‘a’ and ‘b’ represent two distinct layers of cholesterol staining. Fluorescence intensity analysis at the tegument (locations: anterior #1, #2; posterior #3, #4) of single *z*-sections from the centre of the parasite (lower images) was determined and mean ± s.d. fluorescence of the stained cholesterol calculated (graph; *n* = 20 parasites/treatment). Bars = 25 µm. (*b*) One-day-old somules (approx. 1000/treatment) were left untreated (control, 0 mM) or treated with M*β*CD (1 or 10 mM) for increasing durations. Protein extracts were processed for western blotting and probed with anti-phospho-PKC (Ser^660^), -PKC (Thr^410^), -p44/42MAPK (Thr^202^/Tyr^204^) (ERK1/2), -p38 MAPK (Thr^180^/Tyr^182^), -Akt (Thr^308^) or -PKA (Thr^197^) antibodies. Actin was the loading control. Band intensities were quantified and mean relative change in phosphorylation (bar graphs; ±s.d.; *n* = 3) normalized for actin levels was calculated with respect to controls (0 mM) that were assigned a value of 1 (dotted line). (*c*) One-day-old somules (1000/treatment) were either left untreated, treated with 10 mM M*β*CD for 30 min, or treated then reloaded with 1 mM water soluble cholesterol for 15 min without removal of M*β*CD. Protein samples were processed with anti-phospho antibodies and actin was the loading control. Blots are representative of those from two independent experiments. For all panels, *p ≤ 0.05, **p ≤ 0.01 and ***p ≤ 0.001, when compared with controls.

The cholesterol-rich lipid rafts are important hubs for cell signalling across cellular membranes [21], so we hypothesized that tegument raft disruption would modulate protein kinase activities within the parasite. Methyl-β-cyclodextrin (M*β*CD), which selectively extracts cholesterol from lipid raft membranes [37,38] without affecting parasite viability [39], was therefore used to assess the impact of raft disruption on cell signalling in 1-day-old somules. At 10 mM, M*β*CD visibly reduced cholesterol in the outer somule membrane (figure 4*a*), as determined by staining with the cholesterol probe filipin. Signal quantification revealed that after 60 min exposure, 1 mM M*β*CD caused a 10% depletion (*p* ≤ 0.01) of surface membrane cholesterol from the anterior region of the somule, with 10 mM M*β*CD depleting cholesterol by 26% and 21% from anterior and posterior regions, respectively (*p* ≤ 0.001; figure 4*a*). Quantification of absolute cholesterol levels revealed that the tegument of 1-day-old somules contained 13.1 µg (±2.2 µg; *n* = 5) cholesterol per 1000 somules; this was reduced by 5% and 23% following 30 min treatment with 1 and 10 mM M*β*CD, whereas after 60 min cholesterol was depleted by 10% and 40%, respectively (electronic supplementary material, figure S3).

The effect of raft disruption on receptor binding/distribution was investigated. CLSM revealed that somules bound less ligand across their surface following 10 mM M*β*CD treatment. EGFR binding/expression was noticeably reduced, especially visible on maximum projection images, compared to IR binding (electronic supplementary material, figure S4). By contrast, somules not treated with M*β*CD displayed typical EGFR and IR distributions (electronic supplementary material, figure S4, and cf. figure 2). These results suggest that M*β*CD disrupts raft domains leading to the loss of EGFR and IR from the somule surface, or a reduced capacity to engage the EGF/insulin ligand.

To investigate in detail downstream responses of raft disruption on molecular signalling, we evaluated the effect of M*β*CD on the activation of a panel of protein kinases: PKC, extracellular signal-regulated kinase (ERK), p38 mitogen-activated protein kinase (p38 MAPK), Akt/PKB and protein kinase A (PKA), using anti-phospho antibodies previously validated in our laboratory to detect only the activated form of these kinases in *S. mansoni* (figure 4*b*) [13,40–44]. Treatment with 1 mM M*β*CD significantly enhanced the phosphorylation (activation) of all the detected PKCs, whereas 10 mM M*β*CD supressed the activation of the 116 kDa isoform (figure 4*b*). While the overall effects of M*β*CD on ERK and p38 MAPK activation were similar, with only 10 mM stimulating activation (at 20 and/or 30 min), the effect on p38 MAPK activation was more striking with 12-fold increased phosphorylation observed at 30 min (*p* ≤ 0.001) (figure 4*b*). Interestingly, 1 mM M*β*CD significantly affected the activation of Akt/PKB at 10 and 20 min (*p* ≤ 0.01), while 10 mM M*β*CD was without effect. Finally, PKA activation was unaffected by M*β*CD treatment (figure 4*b*). The effect of co-treatment with M*β*CD and ligand was next investigated; however, M*β*CD treatment did not significantly enhance or supress any human EGF- or insulin-mediated effect on the phosphorylation of the protein kinases investigated (electronic supplementary material, figure S5).

Cholesterol repletion has been reported in some systems to reverse the effects of M*β*CD [45,46]. Thus, cholesterol depleted somules (10 mM M*β*CD, 30 min) were reloaded with cholesterol using a water-soluble cholesterol/M*β*CD complex (1 mM, 15 min). Such reloading largely reversed the effects of M*β*CD on protein kinase signalling, supporting the finding that the observed modulation of protein kinase signalling in somules was due to cholesterol depletion from the parasite surface membranes (figure 4*c*).

### 2.5. Cholesterol depletion restricts *S. mansoni* somule motility, growth and development, and stem cell proliferation

**Figure 5.**
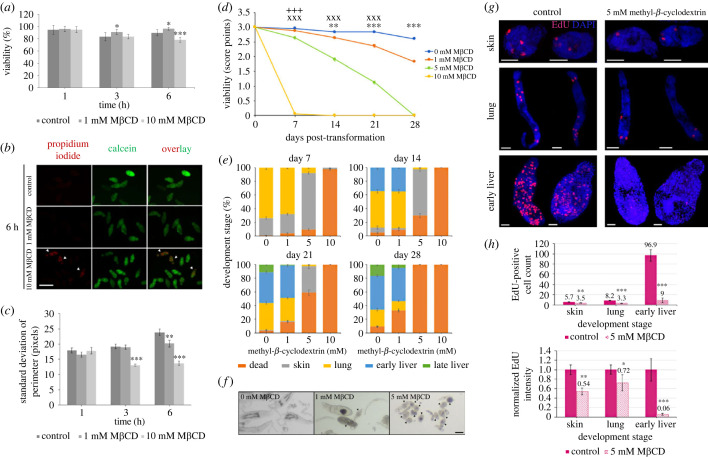
Cholesterol depletion restricts *S. mansoni* somule viability, motility, growth and development, and stem cell proliferation. (*a*) One-day-old somules (1000/well) were left untreated or incubated in M*β*CD (1 or 10 mM) for increasing durations, stained with propidium iodide or calcein, and fluorescence measured. Mean viability (±s.d.; *n* = 6) was calculated from three independent experiments. (*b*) Somules from (*a*) were visualized on a Floid imaging station; green and red fluorescence represents live and dead (arrowhead) parasites, respectively. Images are representative of the total somule populations; bar = 100 µm. (*c*) After treatment, 20 s movies were captured and mean (±s.e.m., *n* = 60) somule motility (standard deviation of the perimeter) determined using ImageJ from two independent experiments. (*d*–*f*) Newly transformed somules were maintained in Basch Medium 169 with 20% human serum and increasing concentrations of M*β*CD. (*d*) Mean viability and (*e*) somule development were evaluated by scoring/staging 30 parasites in each replicate/treatment (±s.e.m., *n* = 450) in three independent experiments, each with five biological replicates. (*f*) Photomicrographs of representative somules were taken at day 21; bar = 100 µm, arrowheads indicate dead somules. (*g*,*h*) Skin, lung and liver somules were left untreated (control) or treated with M*β*CD (5 mM) while being chased with EdU for 24 h. Somules were processed and visualized by CLSM; images (maximum projections of 25 *z*-sections) are representative of those captured from the total parasite populations; bars = 25 µm. (*h*) The mean number of EdU^+^ cells and mean fluorescence intensity (normalized) were determined (±s.e.m., *n* = 10 somules/stage/treatment). *p ≤ 0.05, **p ≤ 0.01 and ***p ≤ 0.001, when compared with controls, except for (*d*) where **p ≤ 0.01, ***p ≤ 0.001 compare between control and 1 mM M*β*CD, ^xxx^*p* ≤ 0.001 between control and 5 mM M*β*CD, and ^+++^*p* ≤ 0.001 between control and 10 mM M*β*CD.

As cholesterol depletion by M*β*CD impacted molecular signalling in parasites, the effect of this compound on short-term somule viability was determined. Using a quantitative dual-fluorescence assay, only 10 mM M*β*CD was found to reduce viability, and only after 6 h treatment (13% reduction; *p* ≤ 0.001; figure 5*a*,*b*). Surprisingly, somules treated with 1 mM M*β*CD showed marginally improved viability at 3 h and 6 h compared to controls (*p* ≤ 0.05; figure 5*a*). Next, the effect of M*β*CD on motility was assessed, using standard deviation of the somule perimeter over 20 s as a proxy for contractile movement. During the first hour, M*β*CD did not impact motility; however, 10 mM M*β*CD supressed motility by 32% at 3 h, with a maximal 42% reduction observed at 6 h compared to controls (*p* ≤ 0.001; figure 5*c*). On the other hand, 1 mM M*β*CD reduced motility (by 15%) only at 6 h (*p* ≤ 0.01; figure 5*c*). These data support that cholesterol depletion from somule surface membranes can affect the viability and motility of the somule and its contractile status.

Using our recently published methods for culturing somules to liver stage using Basch medium plus 20% human serum [47], we next investigated the effect of cholesterol depletion by M*β*CD on longer-term somule survival and development. At 10 mM, M*β*CD was lethal to somules killing almost all by day 7 (figure 5*d*,*e*), while 5 mM M*β*CD killed 33%, 60% and 100% of somules by days 14, 21 and 28, respectively (figure 5*d*,*e*). This 5 mM M*β*CD concentration also restricted the development of surviving somules, with only 8% progressing to lung stage at day 7 compared to 74% in controls (*p* ≤ 0.001; figure 5*e*). Contrastingly, 1 mM M*β*CD only caused increased death at days 21 (16% cf. 3% in control, *p* ≤ 0.01) and 28 (33% cf. 9% in control, *p* ≤ 0.001). The first early liver somules appeared by day 14 and only in control and 1 mM M*β*CD cultures, with no significant difference between these conditions. By day 21, a small proportion (2%) of late liver somules appeared in 1 mM M*β*CD; however, this was considerably less than in controls (*p* ≤ 0.001; figure 5*e*,*f*). By day 28, 7% of the 1 mM M*β*CD-treated surviving somules were late liver stage, compared to 18% in controls (*p* ≤ 0.001; figure 5*e*).

Given that somatic stem cells drive tissue expansion and development of somules to adult worms [48,49], we hypothesized that lipid raft disruption by M*β*CD would blunt stem cell proliferation. First, specificity of the Click-iT Alexa Fluor 594 azide used for staining stem cells in *S. mansoni* somules was confirmed (electronic supplementary material, figure S6). Somules at various developmental stages were then chased with EdU for 24 h in cultures containing a sub-lethal (5 mM) dose of M*β*CD. In controls, typically 5–6 EdU^+^ cells were visible in the posterior half of skin somules, with 8–10 evident within lung somules, and considerably more in early liver somules (figure 5*g*,*h*). Similar size somules were analysed (for each life stage) and area measurements (using ImageJ) were not statistically different between control and treated parasites (mean ± s.d. areas in pixels for control versus treated, respectively: skin, 43 499 ± 4776, 38 187 ± 6683; lung, 27 243 ± 3697, 27 190 ± 10 453; early liver, 48 859 ± 12 090, 43 681 ± 16 830). Following 5 mM M*β*CD treatment, considerably fewer EdU^+^ cells were present in all life stages (*p* ≤ 0.01), with a striking 91% reduction seen in early liver somules when compared with controls (*p* ≤ 0.001; figure 5*g*,*h*). Concomitant with this, the fluorescence intensity of EdU^+^ cells in M*β*CD-treated somules was lower than for control counterparts (*p* ≤ 0.05; figure 5*h*).

Collectively these results demonstrate that M*β*CD is a potent compound that restricts the survival, stem cell proliferation and development of *S. mansoni* somules highlighting the integral importance of cholesterol to these processes.

### 2.6. The EGFR inhibitor CI-1033 restricts *S. mansoni* somule growth, development, and stem cell proliferation, and is lethal at 50 µM

**Figure 6.**
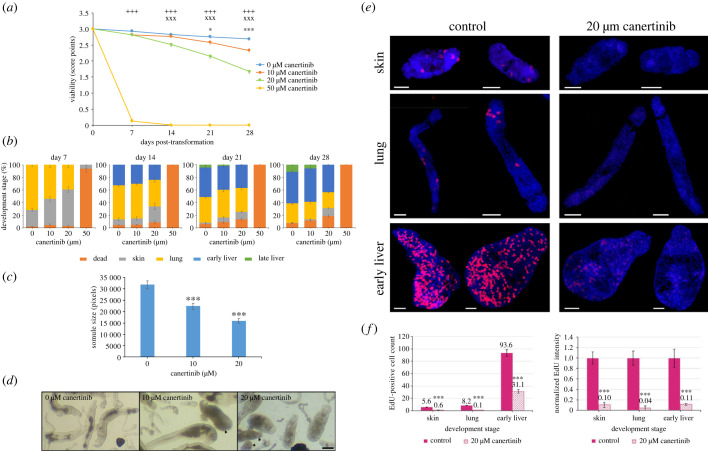
Canertinib restricts *S. mansoni* somule growth, development, stem cell proliferation, and survival. (*a*–*d*) Newly transformed somules were maintained in Basch Medium 169 with 20% human serum and increasing concentrations of canertinib. (*a*) Mean viability and (*b*) somule development were evaluated by scoring/staging 30 parasites in each replicate/treatment (±s.e.m., *n* = 270) in three independent experiments, each with three biological replicates. (*c*) The mean (±s.e.m., *n* = 45) size (area analysis) of early/late liver somules at day 28 was determined using ImageJ. (*d*) Photomicrographs of representative somules were taken at day 21; bar = 100 µm, arrowheads indicate dead somules. (*e*,*f*) Skin, lung and liver somules were left untreated (control) or treated with canertinib (20 µM) while being chased with EdU for 8 h. Somules were processed and visualized by CLSM; images (maximum projections of 25 *z*-sections) are representative of those captured from the total parasite populations; bars = 25 µm. (*f*) The mean number of EdU^+^ cells and mean fluorescence intensity (normalized) were determined (±s.e.m., *n* = 10 somules/stage/treatment). ***p ≤ 0.001, when compared with controls, except for (*d*) where *p ≤ 0.05, ***p ≤ 0.001 compare between control and 10 µM canertinib, ^xxx^*p* ≤ 0.001 between control and 20 µM canertinib, and ^+++^*p* ≤ 0.001 between control and 50 µM canertinib.

Given that EGFRs localize to the parasite surface, we next investigated the effect of CI-1033 (canertinib), a pan-erbB EGFR inhibitor that blocks all members of the ErbB receptor family [50], on somule development and survival. At 50 µM, canertinib was lethal and only 4% of somules survived to day 7. The effects of 10 µM and 20 µM canertinib were less marked, however, with 10 µM only affecting survival from day 21 (*p* ≤ 0.05; figure 6*a*). Importantly, though, all concentrations of the inhibitor restricted somule development. On day 7, lung somules were present in all cultures except for 50 µM canertinib for which survivors were all skin stage; however, in 20 µM canertinib cultures, 32% fewer lung somules were present (figure 6*b*). While somules developed to the late liver stage in control and 10 µM inhibitor treatments (by day 21), somules cultured in 20 µM canertinib failed to develop past the early liver stage (figure 6*b*). Finally, by day 28 there were 55% more dead somules and 5% fewer late liver somules in the 10 µM canertinib cultures (*p* ≤ 0.05) than in controls (figure 6*b*). Somule size analysis, carried out because canertinib did not completely abolish somule development, revealed that somules were 30% and 50% smaller in the 10 µM and 20 µM canertinib cultures, respectively (*p* ≤ 0.001), compared to those grown without the inhibitor (figure 6*c*).

Surprisingly, chasing somules with EdU in the presence of 20 µM canertinib for 24 h caused some somule death, so the assay was done for 8 h. Nevertheless, over only 8 h, 20 µM canertinib almost completely blocked EdU incorporation into stem cells of both skin and lung somules; moreover, a significant 67% decrease in EDU^+^ cell number was observed in early liver somules (*p* ≤ 0.001; figure 6*e*,*f*). Fluorescence intensity analysis revealed that 20 µM canertinib reduced EdU incorporation by 90%, 96% and 89% in skin, lung and early liver somules, respectively (*p* ≤ 0.001; figure 6*e*,*f*). There were no significant differences in somule size between control and treated somules as assessed by ImageJ (mean ± s.d. areas in pixels for control versus treated, respectively: skin, 48 466 ± 14 054, 51 133 ± 8655; lung, 28 160 ± 8027, 27 958 ± 5060; early liver, 30 175 ± 2985, 32 017 ± 8298).

These findings demonstrate that the EGFR inhibitor canertinib restricts the survival, stem cell proliferation and development of *S. mansoni* somules and that this is likely mediated through EGFRs expressed at the parasite surface. Signalling through the lipid rafts probably mediates these processes.

## 3. Discussion

**Figure 7.**
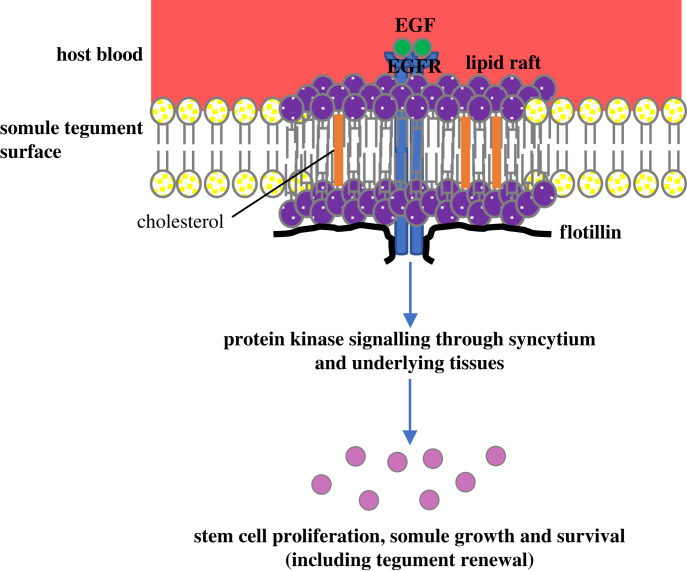
Proposed model illustrating the involvement of lipid rafts in *S. mansoni* stem cell proliferation, somule growth and survival. Host growth factors, such as EGF, bind to cognate receptors (e.g. EGFRs) that localize to cholesterol-rich lipid rafts at the parasite surface; EGFRs may move in or out of the rafts according to their activation status. Downstream protein kinase (e.g. ERK) signalling is next activated that transmits signals through the syncytial layer and underlying tissues. These signals promote somatic stem cell proliferation, growth and development, and survival of the somules. We hypothesize that this process also maintains the tegument through activation of the stem cells that underly the tegument and are important for its renewal. Signalling to internal structures may also be transmitted through the extensive nervous tissue (not shown).

The ability of the schistosome surface membrane to interact with, and respond to, human growth factors is likely to underpin processes that drive parasite success in the definitive host; however, such phenomena have not been extensively explored. The findings of this study support the hypothesis that human host growth factors can interact with the surface of schistosomes, signal through lipid rafts, and promote stem cell proliferation, growth and development of the parasite (figure 7). Our data thus uncover novel aspects of schistosome–host interplay and schistosome cellular biology that could fundamentally support parasite establishment and survival within the host.

One of the most important changes in terms of host–parasite communication and schistosome protection during cercaria to somule transition is the complete replacement and remodelling of the tegument surface membrane [51]. The tegument is a dynamic organ with constant renewal of composition, proteins appearing and disappearing at its surface as somules develop [11]. In this context, we discovered that the ability of freshly prepared somules to respond to EGF and form lipid rafts was dynamic, with GM1-clustering becoming more prominent as the somules developed over 24 h. Moreover, the rafts appeared in an anterior to posterior fashion during somule development but maintained a preferential anterior localization throughout. Fluorescent analogues of human EGF and insulin bound the surface of the developing somule in a similar fashion, and co-localization with raft microdomains was demonstrated by CLSM. Although indirectly detected, we consider binding sites to represent EGFR and IR hotspots, respectively, at the parasite surface, particularly given that three EGFRs and two IRs exist in the parasite [32,33], with the schistosome IRs known to be able to bind human insulin [52,53]. Given that the anterior cone is the first part of the parasite to breach the skin, and that correct orientation in the dermis might be important to parasite survival [54,55], we speculate that the predominant anterior location of the EGFR receptors/rafts facilitates the navigation of the somule through the skin layers enabling location and penetration of capillaries. If correct, then our findings could support future work to answer long-standing unanswered questions concerning how schistosomes navigate within their hosts.

To better characterize the molecular composition of *S. mansoni* lipid rafts, we undertook the first proteomic analysis of detergent soluble and insoluble (DRM) membrane components in schistosomes. We identified 41 proteins present exclusively in the flotillin-resident ‘raftophilic’ (TI) DRM fraction, with an additional 133 proteins shared with the TS fraction. Selected TI proteins are considered here in the context of parasite biology in the definitive host. (i) The LDL receptor, also raft-associated in human mesenchymal stem cells [56], that can bind human LDL [57]. Because schistosomes cannot synthesize their own sterols or fatty acids, they must obtain them from the host [58]; although LDL uptake across the *S. mansoni* tegument has not been definitively reported, it cannot be ruled out *a priori* and is worthy of investigation. (ii) Calmodulin, a highly conserved Ca^2+^ sensing protein, also detected in lipid raft fractions of human cells [56,59], that has been shown to be important to schistosome larval development [60]. An important partner of calmodulin, calmodulin-dependent protein kinase II (CaMKII), has recently been shown to regulate neuromuscular behaviour and survival of *S. mansoni* [61]. (iii) Annexins, members of the Ca^2+^/lipid-binding protein family, shown to be associated with lipid rafts in human cells [62], and upregulated at the apical membrane of somules during development [11]. These proteins are thought to facilitate attachment of the tegument membranocalyx to the plasma membrane [63] and associate with schistosome extracellular vesicles (EVs) [64,65] that might play a role in host–parasite communication [66]. Several other proteins recovered in the TI fraction including (iv) histones, (v) GTP-binding nuclear protein (Ran), (vi) dynein light chain, (vii) Fer-1 related, intermediate filament protein, (viii) thioredoxin and (ix) high mobility group B1, were also identified in EV proteomic analyses [64–66]. (x) Putative synaptotagmin, a vesicle trafficking protein, was also present in the TI fraction. Thus, we propose that lipid rafts represent important structural/functional elements of schistosome EVs, that might be crucial either for the biogenesis (e.g. acting as sorting platforms) and maintenance of EVs, or their functional interactions with host cells. Finally, the specific occurrence of several cytoskeleton-associated/linker proteins in the TI fraction, including (xi) putative myosin light chain 1, (xii) putative nebulin, (xiii) Eps8-related protein and (xiv) protein 4.1 G, provides evidence for the concept that components of the cytoskeleton can localize to lipid rafts providing platforms for cytoskeletal tethering and communication to the extracellular space [67] in somules, which could occur via (xv) putative claudin, a protein also identified in the TI fraction.

Importantly, we also demonstrate that protein kinase signalling within *S. mansoni* can be modulated by cholesterol depletion, with contrasting activation/inhibition of PKC seen with low (1 mM) and high (10 mM) M*β*CD doses, respectively. ERK, and particularly striking p38 MAPK, hyperactivation were also seen. The modulatory effects were reversed by cholesterol repletion, and the M*β*CD concentrations/exposure durations used did not impair somule motility or viability, giving assurance for a specific effect of M*β*CD on tegument cholesterol. Collectively, our results suggest that the proper functioning of certain signalling systems in *S. mansoni* somules requires the integrity of lipid raft microdomains, as has been demonstrated using cholesterol extraction for proteins like PKC [68] (inhibition), ERK [69–71] (hyperactivation), p38 MAPK [72–74] (hyperactivation) and Akt [75] (cell type specific activation/inhibition) in mammalian cells. Such modulatory effects are considered to be dependent upon interaction of the kinase, upstream activators, or other important interactor proteins with the membrane rafts, in a pathway specific fashion, and can be unaffected by ligand interaction as shown for *S. mansoni* here [67–75]. Importantly, we have shown previously that EGF and insulin stimulate ERK phosphorylation in *S. mansoni* somules and, together with others, have shown PKC, ERK and p38 MAPK to be implicated in regulating various fundamental schistosome processes including movement, survival, worm maturation, development and egg production [31,41,42,76–78]. Considering that lipid raft modification led to modulation of several protein kinase pathways, the importance of these microdomains to somule motility and short-term viability was investigated, revealing dose- and time-dependent effects, with significant declines in motility and viability seen at 3 h and 6 h, respectively. Moreover, the findings detailed here, when coupled with our recent discovery that Akt regulates glucose transporter 4 (SGTP4) expression at the schistosome tegument [13], indicate that lipid rafts might play a vital role in glucose import in the parasite, possibly in response to host insulin.

Investigation into the effects of lipid raft disruption by M*β*CD, and the EGFR inhibitor canertinib, on long-term somule survival and growth/development revealed that both compounds potently restricted parasite longevity and development. Concomitant with this, M*β*CD and canertinib blocked the proliferation of somatic stem cells in skin, lung and liver somules, with particularly compelling suppression seen with the EGFR inhibitor. These somatic stem cells that are like neoblasts of planarians [79], non-parasitic relatives of schistosomes, can self-renew and differentiate and are thought to support schistosome growth and longevity in immunocompetent hosts such as humans [48,79]; the stem cells are also crucial for replacing the parasite tegument [49,80]. Even though the mechanisms governing stem cell proliferation are largely unknown in schistosomes, with EGFR being identified as a likely regulator here, a growing body of evidence from other organisms, including invertebrates, supports that EGFRs might play a conserved role in stem cell proliferation in general [81–84]. In a cell more than 50% of the EGFRs are speculated to be concentrated in lipid rafts [85], highlighting the likely importance of these microdomains to stem cell proliferation in *S. mansoni* and supporting the congruence between our M*β*CD and canertinib EdU^+^ cell data. Furthermore, the increased phosphorylation of PKC and ERK in the *S. mansoni* somule tegument during early development, in response to human EGF, insulin or IGF-1 [31], could be linked to the early transformation of somules that is thought to occur via activation of downstream PKC and ERK signalling pathways. In the current work, we did not determine the effect of the EGFR inhibitor canertinib on downstream protein kinase activation or lipid raft–EGF/EGFR interactions/localization. Such experiments would be worthwhile, however, particularly employing the different somule life stages and different canertinib concentrations, as they may shed further light on the links between EGFR signalling and downstream signalling/functional processes in the parasite. Nevertheless, taken together, the results of this study suggest that stem cell proliferation in *S*. *mansoni* depends on signalling by extracellular host cues, including EGF, mediated through/by lipid rafts, which in turn drives schistosome growth and development.

Despite characterizing a novel mechanism that supports the growth, development, and survival of *S. mansoni* somules, and proposing a working model (figure 7), several questions remain unresolved. Firstly, we failed to detect EGFRs and IRs in our proteomic fractions, although EGF receptor kinase substrate eps8-related (Smp_035260), an important EGFR partner [86], was present in the TSI fraction. We surmize that this is most likely due to relatively low expression of the receptors and/or that more protein is required for analysis; we resolved 41 proteins in our DRM analysis, but proteomic studies of lipid rafts in human cells typically reveal hundreds of proteins [87,88]. Nevertheless, the EGFRs (Smp_344500, Smp_165470, Smp_093930) and IRs (Smp_341160, Smp_009990) are expressed by somules (electronic supplementary material, figure S2*a*), and by the neoblast cells and tegument progeny in adult *S. mansoni* for which single cell expression data are available (electronic supplementary material, figure S2*b*). Our future work aims to characterize the lipid raft proteome in *S. mansoni* more comprehensively. Secondly, it is not known the extent to which human-derived EGF and -insulin are vital to the parasite *in vivo*, particularly given that schistosomes also express insulin-like peptides endogenously [89]. Thirdly, it is unclear how signals received at the somule surface and communicated through the lipid rafts might regulate stem cell proliferation deep within the somule tissue. Although we hypothesize that these signals are propagated across the tegument syncytium and communicated to underlying tissues (figure 7), it is plausible that the extensive nervous tissue of the parasite plays an important role in distributing host signals throughout the parasite.

The requirement for certain host-derived molecules for the growth and development of schistosomes in the human host has been known for several decades, particularly highlighted by the comprehensive, ground-breaking *S. mansoni* culture work of Paul Basch (reviewed in [90]). By studying *S. mansoni* in the context of growth factor interactions with the parasite tegument, we discover that cholesterol-rich lipid raft domains and EGFRs are crucial for stem cell proliferation, growth, development and survival of the somule, an early parasite life stage that is generally refractory to the only drug deployed for control, praziquantel [91]. Our findings thus offer valuable and novel insights into the mechanisms governing schistosome vitality that might be exploitable for the future control of human schistosomiasis.

## 4. Material and methods

### 4.1. Parasites and parasite culture

Livers from mice, experimentally infected with *S. mansoni* (Puerto Rican strain), were from Dr Gabriel Rinaldi, Wellcome Sanger Institute (Cambridge, UK). Laboratory animal use was within designated facilities regulated by and complying with the UK Animals (Scientific Procedures) Act 1986 and infection protocols were approved by the Wellcome Sanger Institute Animal Welfare and Ethical Review Body in accordance with the UK Home Office project licence P77E8A062 (Gabriel Rinaldi). The infected livers were homogenized and *S. mansoni* eggs isolated, miracidia hatched in spring water, and *B. glabrata* (approx. 10 mm diameter) exposed to 20 miracidia/snail in 24-well plates (Nunc) overnight at 26°C. Infected *B. glabrata* were also supplied by the Biomedical Research Institute (BRI; Maryland, USA) via the National Institute of Health–National Institute of Allergy and Infectious Disease (NIH-NIAID) Schistosomiasis Resource Centre under NIH-NIAID contract HHSN272201000005I. Snails were housed in plastic boxes containing water filtered through a Brimak carbon filter (Silverline UK) in an incubator maintained at 26°C with a 12 h : 12 h light : dark cycle. When patent, snails were placed under a light and emergent cercariae collected and transformed mechanically into skin somules using our standard approaches [44]. The somules were then loaded into a 48-well tissue culture plate (Nunc; 1000 somules ml^−1^ well^−1^) in Basal Medium Eagle (BME) containing 200 U ml^−1^ penicillin, 200 µg ml^−1^ streptomycin and 500 ng amphotericin B and incubated at 5% CO_2_/37°C for 18–24 h (1-day-old somules). For growth/developmental studies, somules were instead cultured in Basch Medium 169 [92] with antibiotics/antimycotics and 20% human serum as per our published protocol [47].

### 4.2. Lipid raft staining

The Vybrant Alexa Fluor 488 lipid raft staining kit (Invitrogen) was used to stain rafts. Somules, 0–30 min, 1 h/3 h post-transformation, or cultured in BME overnight, were left untreated or exposed to human EGF (Life Technologies 10605HNAE25; 15 ng ml^−1^; 5–30 min) in culture plates at ambient. Plates were next placed on ice, media/EGF removed, somules washed briefly with 500 μl chilled BME, and incubated in Alexa Fluor 488 CT-B conjugate (1 µg ml^−1^, 10 min) on ice to label ganglioside_GM1_. After three washes in phosphate buffered saline (PBS), somules were incubated in anti-CT-B antibody (15 min) on ice to crosslink the CT-B conjugate for raft clustering. Somules were further washed in PBS and fixed (4% paraformaldehyde, 30 min, room temperature (RT)). Washing was repeated, somules transferred to silane-prep slides (Sigma), slides dried at 60°C, and the parasites mounted in Vectashield (Vector Laboratories) antifade medium under coverslips. Specimens were visualized on a Leica SP2 AOBS CLSM (40× or 63× oil immersion objectives) and images captured; similar photomultiplier tube (PMT) voltages/laser settings were maintained for all samples. Fluorescence intensity was determined at four randomly selected points on the tegument of each somule within a *z*-scan using Leica quantification software by constructing digital lines (one/two anterior, one/two posterior) and generating graphs of intensity across each line. Peak intensity at the tegument intersection was noted; 20 somules per treatment were analysed.

### 4.3. Analysis of EGF and insulin binding to somules

One-day-old somules were stained with Alexa Fluor 488-conjugated human EGF (2 µg ml^−1^; Invitrogen E13345) or FITC-insulin human (1 µM; Sigma I3661) in BME for 5–30 min, BME was removed, and parasites fixed in 4% paraformaldehyde (30 min, RT) followed by extensive washing in PBS. For Alexa Fluor 488-conjugated EGF only, parasites were permeabilized (0.3% Triton X-100, 1 h), washed thrice 20 min each with PBS, and incubated in rhodamine phalloidin (0.2 µg ml^−1^) overnight at 4°C with agitation. Following three 20 min washes, parasites were mounted on silane-prep slides in Vectashield. For investigating EGFR and IR expression on transforming somules, parasites were stained for 5 min at various time points (10–30 min, 1, 3 and 24 h) post-transformation in BME with the fluorescent ligands and processed. Parasites were visualized and images captured by CLSM, and fluorescence intensity at eight randomly selected points (20 somules per treatment) determined. Ligand competition was also done to determine specificity of binding; somules were incubated with EGF (10 µg ml^−1^; Life Technologies 10605HNAE25) or insulin (50 µM, Tocris 3435) for 15 min prior to staining with the equivalent fluorescent ligands and were processed.

To demonstrate co-localization of receptors with lipid rafts, dual staining of ganglioside_GM1_ and EGFRs/IRs was performed. Somules were stimulated with EGF (15 ng ml^−1^), stained with Alexa Fluor 594 CT-B conjugate and cross-linked with anti-CT-B antibody (§4.2). All washes were done in chilled BME to prevent potential PBS interference with receptor staining. Somules were then incubated with either Alexa Fluor 488-conjugated EGF (2 µg ml^−1^) for 5 min or FITC-insulin (1 µM) for 30 min, fixed in 4% paraformaldehyde and processed for CLSM.

### 4.4. Immunofluorescence staining and co-staining of lipid rafts and human ligands

Somules were fixed in 4% paraformaldehyde, washed with PBS, transferred to 48-well tissue culture plates and incubated with 1% glycine for 15 min, and permeabilized (0.3% Triton X-100 in PBS, 1 h). After three 10 min washes in PBS, somules were blocked with 10% normal goat serum (Invitrogen) for 2 h, re-washed, and incubated in primary antibody (anti-flotillin-1 (BD Biosciences, 610820), -Gq/II*α* (Millipore, 06-709), -ras (CST, 3965), each at 1 : 50 in 1% (w/v) BSA in PBS) for 3 days at 4°C with agitation. Next, parasites were washed three times 30 min each and incubated in Alexa Fluor 488 secondary antibodies (1 : 500 in PBS; Thermo Fisher, A11008, A11001), and 0.2 µg ml^−1^ rhodamine phalloidin (where appropriate), for 2 days at 4°C. After extensive washing (three times 30 min) with PBS, somules were mounted onto silane-prepared slides and visualized by CLSM. Images were captured using identical laser settings to those for negative control (without primary antibody) samples. For flotillin/lipid raft co-localization, somules cultured overnight in BME were first stimulated with EGF (15 ng ml^−1^) for 5 min, stained with Alexa Fluor 594 CTB conjugate and cross-linked with anti-CT-B antibody prior to immunohistochemistry.

### 4.5. Isolation of DRMs and proteomic analysis using LC-MS/MS

DRMs were prepared as detailed by Adam *et al*. [93] from somule teguments obtained by freeze fracture [13,94]. One-day-old somules (approx. 25 000) were treated with EGF (15 ng ml^−1^) for 5 min, placed on ice (5 min), centrifuged (12 100×*g*) for 30 s, and pooled together on ice in 100 µl PBS prior to plunging in liquid nitrogen for 15 min. Somules were thawed, placed on ice, washed gently with 1 ml ice-cold PBS and pulse-vortexed 20 times, 1 s each, and centrifuged (500×*g*) in a Hettich Universal 32R centrifuge (1689-L rotor) for 5 min at 4°C to pellet somule bodies. The supernatant containing the parasite surface membranes and syncytial cytoplasm was centrifuged at 16 000×*g* for 10 min at 4°C and the supernatant collected as ‘cytosolic’ fraction. The high-speed pellet was resuspended in Buffer ‘A’ (25 mM MES, 150 mM NaCl, pH 6.5) and combined with an equal volume of Buffer ‘B’ (25 mM MES, 150 mM NaCl, pH 6.5, 2% Triton X-100), incubated on ice for 60 min and centrifuged at 16 000×*g* (20 min, 4°C). The supernatant was the TS fraction; the pellet was rinsed with Buffer ‘A’ and reconstituted in Buffer ‘C’ (10 mM Tris–HCl, pH 7.6, 150 mM NaCl, 60 mM β-octylglucoside) giving the TI fraction. A 50 µl aliquot of the ‘cytosolic’ fraction and the total TI/TS fractions were heated in LDS sample buffer with reducing agents and processed for western blotting with anti-flotillin-1, anti-Ras, and anti-β-tubulin (Cell Signalling Technologies (CST), 2128) antibodies.

Approximately 150 000 1-day-old somules were used for lipid raft proteomics. Samples were prepared as above but β-octylglucoside, which is incompatible with MS, was excluded from Buffer ‘C’. The resultant pellet was rinsed with Buffer ‘A’, LDS sample buffer added, and TS and TI samples heated at 95°C, loaded onto gels and electrophoresed into the gel (for approx. 1 cm; 180 V approx. 6 min). After electrophoresis, the gel was rinsed three times 5 min each with 200 ml ultrapure water. The gel was stained with GelCode Blue and the region containing protein excised and sent for LC-MS/MS at the Proteomics laboratory, University of York, using their standard protocols. Searches were performed against *S. mansoni* genomic database (11 723 sequences; 5 597 432 residues) using a local copy of the Mascot program (version 2.5.1; Matrix Science) with enzyme specificity of trypsin, fixed modification of carbamidomethyl, variable modifications of oxidation and deamidation, peptide tolerance of 3 ppm and mass tolerance of 0.5 Da. The results obtained were imported into Scaffold (version 4.7.5; Proteome Software) and further searched against the same databases using the X!Tandem. Protein identifications were accepted if they had maximum protein/peptide false discovery rate of 1% and at least two unique peptide identifications per protein. Proteins encompassing similar peptides, but which could not be distinguished based on MS/MS analysis alone, were categorized to fulfil the principles of parsimony whereas proteins sharing significant peptide evidence were categorized into clusters.

### 4.6. Disruption of lipid rafts using M*β*CD

One-day-old somules were incubated with 1 or 10 mM M*β*CD (or water, control) in BME for 30 and 60 min. Culture plates with somules were placed on ice, media removed, and stained with filipin as described previously [95]. Briefly, somules were fixed in 4% paraformaldehyde for 30 min, washed thrice with PBS, and incubated with 50 µM filipin III (filipin; Tocris 6250) or DMSO (control) in PBS for 1 h at RT followed by four PBS washes. Somules were mounted on silane-prep slides in Vectashield and were visualized by CLSM. For analysis, *z*-scans through the centre of 20 somules were captured maintaining similar PMT voltages and laser settings for all samples. Images were analysed using Leica quantification software to determine the fluorescence intensity at four randomly selected points on the tegument with and without treatment.

The cholesterol content of the somule tegument was determined using an Amplex Red cholesterol assay kit (Invitrogen) according to the manufacturer's instructions. One-day-old somules (approx. 20 000 per treatment) were either untreated (control) or treated with M*β*CD (1 mM or 10 mM in BME; 30 and 60 min). Somules were transferred to cooled microfuge tubes and centrifuged at 12 100×*g* for 30 s; pellets were washed thrice with PBS, centrifuging between each wash. Somules were then pooled and resuspended in 100 µl PBS. Somule teguments were isolated by freeze-fracture (§4.5) and samples reconstituted in reaction buffer to a final volume of 50 µl. Following this, 50 µl cholesterol reference standards and samples were transferred to a black-walled 96-well plate (Nunc) and 50 µl Amplex Red reagent/HRP/cholesterol oxidase/cholesterol esterase working solution added. Reactions were performed for 3 h at 37°C with fluorescence measured on a FluorStar Optima plate reader (BMG Labtech; excitation 544 nm, emission 590 nm). Cholesterol content (μg) of the tegument per 1000 somules was calculated.

To establish the effect of raft disruption on lipid raft staining and human ligand binding, 1-day-old somules were incubated with M*β*CD (1 mM or 10 mM) or water (control) in BME for 30 min. For lipid raft localization, somules were exposed to 15 ng ml^−1^ EGF for 5 min prior to lipid raft staining (§4.2). To examine the localization of receptors after raft disruption, M*β*CD treated/untreated somules were incubated with either Alexa Fluor 488-conjugated EGF (2 µg ml^−1^; 5 min) or FITC-insulin (1 µM; 30 min). Somules were mounted on silane-prep slides in Vectashield and somules visualized by CLSM.

### 4.7. Effects of raft disruption on protein kinase signalling

Lipid rafts were disrupted by cholesterol depletion to assess its effect on the basal activities and stimulatory responses of protein kinase pathways. One-day-old somules were treated with either water (control) or M*β*CD (1 mM or 10 mM) in BME for increasing durations (0–30 min). Somules were transferred to chilled microfuge tubes on ice and processed for western blotting with anti-phospho antibodies (§4.8). Furthermore, somules were exposed to human insulin (1 µM; 10 or 30 min) or EGF (15 ng ml^−1^; 30 min) following raft disruption with M*β*CD (1 mM or 10 mM) for 30 min and processed for western blotting. Finally, somules were exposed to 1 mM cholesterol water-soluble form (cholesterol/M*β*CD complex) for 15 min following treatment with 10 mM M*β*CD (or water, control) for 30 min and processed for western blotting to evaluate the effect of cholesterol replenishment.

### 4.8. SDS-PAGE and western blotting

After treatment, somules were chilled on ice (5 min), pulse centrifuged (12 100×*g*) and lysed with LDS sample buffer/reducing agent (Invitrogen). Samples were then heated (95°C, 5 min), sonicated (30 s), and lysates electrophoresed with SeeBlue pre-stained markers on Bolt Bis-Tris Plus gels with MES/SDS buffer system (Life Technologies). Proteins were semi-dry transferred to nitrocellulose, blots stained with Ponceau S to confirm homogeneous transfer and blocked for 1 h in 1% bovine serum albumin (BSA) or 5% non-fat dried milk (for flotillin-1). After washing in tween tris-buffered saline (TTBS), blots were incubated with either anti-phospho-p38 (Thr^180^/Tyr^182^), -ERK (p42/p44 MAPK) (Thr^202^/Tyr^204^), -PKA-C (Thr^197^), -PKC (pan) (*β*II Ser^660^), -PKC (pan) (ς Thr^410^), or -Akt (Thr^308^) antibodies (CST, 9215, 9101, 4781, 9371, 2060, and 2965, respectively), or anti-flotillin-1, -Gq/II*α*, -ras, and β-tubulin, each at 1 : 1000 (or 1 : 500 for Ras) in 1% BSA. Blots were next washed in TTBS and incubated in horseradish peroxidase (HRP)-conjugated anti-rabbit or anti-mouse secondary antibodies (CST; 1 : 3000, 2 h) and incubated in West Pico (Pierce) or ECL Prime (GE Healthcare) detection reagents (depending on required sensitivity) and bands visualized using a GeneGnome (Syngene) imaging station. Blots were stripped in Restore western blot stripping buffer (Thermo Scientific) for 3 h and incubated in an alternative primary antibody or HRP-conjugated anti-actin (1 : 3000) antibodies to determine total protein loading. Relative band intensities were quantified using Gene Tools and phosphorylation levels normalized according to actin intensity.

### 4.9. Effect of M*β*CD on short-term somule viability and motility

One-day-old somules were treated with either M*β*CD (1 or 10 mM) or water (control) in BME for 1, 3 and 6 h, transferred to microfuge tubes and centrifuged at 200×*g* for 15 s. Somules were washed once (1 ml) with, and resuspended in (100 µl), warmed PBS (37°C). For negative controls, somules were centrifuged and killed by heating at 65°C for 10 min. Next, somules were stained using a Cell Health kit (CST; 13837); 100 µl labelling solution was added giving final concentrations of 1 µM calcein-AM and 1.5 µM propidium iodide (PI). Somules were transferred to black-walled 96-well microtitre plates and incubated for 10 min (5% CO_2_/37°C) to ensure esterase conversion of non-fluorescent calcein-AM to the fluorescent form within live parasites. To compensate for possible inter-plate differences, a blank was set up with labelling solution only. Fluorescence was measured using a FluorStar Optima reader (at 37°C)—PI/dead (544 nm excitation/612 nm emission) and calcein-AM/live (485 nm excitation/520 nm emission)—and viability (%) calculated as in Peak *et al*. [96]. Next, approximately 100 somules from each treatment were transferred to a cavity slide and somule images captured using a Floid cell imaging station (ThermoFisher Scientific).

For motility assays, 1-day-old somules (approx. 500 per well) were incubated with either water (control) or M*β*CD (1 or 10 mM) in BME. Movies (each 20 s) of somules were captured at 1, 3 and 6 h using a Moticam 1080 digital camera and Motic SMZ171 stereomicroscope. Behavioural effects of raft disruption on somule movement were quantified using wrMTrck for ImageJ [97] (http://www.phage.dk/plugins/wrmtrck.html) after movies were converted to .avi using ffmpeg (https://brew.sh/). Frames were converted to greyscale, background subtracted, Otsu threshold adjusted to best detect the somules present, and wrMTrck selected for analysis. Standard deviation of the perimeter was used as a proxy for movement as it enables evaluation of somule contractions and distensions in response to treatment [31]. Somules touching each other were manually excluded from the analysis.

### 4.10. Effect of M*β*CD and canertinib on long-term somule viability, development and stem cell proliferation

We employed our recently published methods for culturing somules to liver stage using Basch Medium 169 plus 20% human serum [47]. To assess the effect of cholesterol depletion or EGFR inhibition, various concentrations of M*β*CD (1–10 mM) or CI-1033 (canertinib dihydrochloride, Tocris 5916, 10–50 µM), respectively, were added to the media with water as controls. Somules were maintained at 37°C/5% CO_2_ for the duration of the assay and three independent experiments were performed for each condition. Media were changed every 3 days, each time replenishing with fresh M*β*CD or canertinib.

Scoring (0–3) for somule viability was based on standard procedures for compound screening at the WHO-TDR [98–100], where: 0 = dead parasites, severe granulation, no contractile movement; 1 = medium granulation, limited contractile movement; 2 = slight granulation, slowed contractile movements; 3 = totally vital, no granulation, normal regular contractile movements. Skin somules were identified as those parasites that retained the shape of the cercarial head, whereas lung somules were characteristically more elongated; early liver somules were those larger in diameter/length with a clearly observable gut, whereas the late liver somules possessed clearly visible oral and ventral suckers with increased body length posterior to the ventral sucker [92]. Somules were scored and developmentally evaluated weekly and images of somules were captured using a Moticam 1080 digital camera and a Motic SMZ171 stereomicroscope. To evaluate the impact of canertinib on the size of liver somules, images were captured and somule size determined using the measurement function in ImageJ and mean somule size in pixels calculated.

To investigate the effect of cholesterol depletion and EGFR inhibition on stem cell proliferation in somules, Click-iT EdU with Alexa Fluor 594 azide (Thermo Fisher Scientific) was used according to described protocols [101,102] and manufacturer's instructions. All stages of somules were washed briefly in warmed BME and were transferred to individual wells of a 96-well tissue culture plate (Nunc) with 100 µl warmed BME supplemented with antibiotics/antimycotics. Next, 100 μl BME containing 20 µM EdU was added to each well. To evaluate the effect of cholesterol depletion, somules were chased for 24 h (37°C/5% CO_2_) with 5 mM M*β*CD (or water, control), whereas for EGFR inhibition, somules were chased for 8 or 24 h (at 37°C/5% CO_2_) with 20 µM canertinib (or water, control). After the chase, media were removed and somules fixed in 200 μl PBST (4% paraformaldehyde, 0.3% Triton-X-100, in PBS) for 4.5 h at RT. The fixative was removed and somules were dehydrated in 50% MeOH followed by 100% MeOH, before placing at −20°C (in 100% MeOH). The next day, somules were rehydrated with 50% MeOH for 10 min, washed with PBST, and treated with proteinase K (6 µg ml^−1^ in 29.5 mM Tris HCl, pH 8.0) for 25 min at RT, before a final PBST incubation for 1 h. Somules were then washed briefly with 3% BSA in PBS before being incubated in 100 µl Alexa Fluor 594 reaction cocktail (100 µl reaction buffer, 100 µl buffer additive, 800 µl copper sulfate) for 3 h at RT in the dark. Finally, somules were washed twice, 15 min each, with 3% BSA in PBS, transferred to silane-prep slides and mounted with SlowFade gold antifade mountant with DAPI (Invitrogen).

Maximum projections of each somule stage for each treatment were captured by CLSM and EdU^+^ proliferating cells manually counted on at least 10 somules (skin to early liver stages) per treatment. The fluorescence intensity of EdU^+^ cells was also determined, using ImageJ. Regions of interest (containing only the EdU^+^ cells) and the entire somule (for parasite size analysis) were selected and three parameters recorded: area of EdU^+^ cell(s) and parasites, mean grey value, and integrated density, the latter of which was used as a measure of overall fluorescence intensity. To compensate for different intensities at different somule stages, net average fluorescence intensity values were normalized to each corresponding control value that was designated 1.

### 4.11. Bioinformatics and statistical analysis

Analysis of EGFR and IR gene expression in somules and other life stage/organs was performed using Schisto.zyx (www.meta.schist.zyz), using EGFR and IR ‘Smp_’ identifiers. The normalized expression data were categorized into decile ranges, with the greatest expression level assigned a value of 100%. Cell type gene expression maps were generated for each EGFR and IR Smp identifier using SchistoCyte Atlas (www.collinslab.org/schistocyte/). Where appropriate, statistical analysis was done with one-way or two-way analysis of variance (ANOVA) using Minitab (version 19); comparison of sample means was then performed using Fisher's *post hoc* multiple pairwise comparison test.

## Data Availability

The data analysed during this study are included in the paper, and in the electronic supplementary material files [103].
